# Impact of Biologics and Proton Pump Inhibitors on Gastrointestinal Infection Risk in Inflammatory Bowel Disease Patients: A Retrospective Analysis of Pathogen-Specific Outcomes and Treatment Interactions

**DOI:** 10.3390/biomedicines13071676

**Published:** 2025-07-08

**Authors:** Ryan Njeim, Elie Moussa, Chapman Wei, Joelle Sleiman, Reem Dimachkie, Liliane Deeb

**Affiliations:** 1 Department of Medicine, Staten Island University Hospital, Staten Island, NY 10305, USA; emoussa@northwell.edu (E.M.); cwei4@northwell.edu (C.W.); jsleiman@northwell.edu (J.S.); 2 Department of Medicine, Division of Gastroenterology and Hepatology, Northwell Staten Island University Hospital, Staten Island, NY 10305, USA; rdimachkie@northwell.edu (R.D.); ldeeb1@northwell.edu (L.D.)

**Keywords:** inflammatory bowel disease, biologic therapy, proton pump inhibitors, gastrointestinal infections, clostridioides difficile

## Abstract

**Background/Objectives**: Inflammatory bowel disease (IBD) patients face elevated gastrointestinal (GI) infection risks due to immune dysregulation and gut dysbiosis. While steroids and immunosuppressants are known to increase infection risk, data on biologics and proton pump inhibitors (PPIs) remain limited, particularly for non-Clostridioides difficile (C.diff) infections. **Methods**: This retrospective cohort study analyzed 9849 hospitalized IBD patients (2013–2023) from the Northwell Inpatient Database. Patients were categorized into four groups: biologics-only, PPIs-only, both, or neither. GI infections were identified via C.diff PCR, GI PCR, and chart review. Multivariate logistic regression adjusted for demographics, BMI, and IBD type. **Results**: GI infections occurred in 1.75% of patients, with significantly higher odds in those on biologics alone (OR 21.5, 95% CI 11.7–39.4) or with PPIs (OR 16.6, 95% CI 10.2–26.8) versus untreated patients. Non-C.diff infections were strongly associated with biologics (OR 20.7, 95% CI 10.2–41.9). PPIs alone increased slightly the risk of GI infections (OR 1.6, 95% CI 1.1–2.4). Vedolizumab and adalimumab had the highest infection risks among biologics (26.8% and 22.7%, respectively). Bacterial pathogens, such as *E. coli* and *Salmonella*, predominated, with no significant difference in causative agents across treatment groups. **Conclusions**: Biologic therapy greatly increases GI infection risk in IBD patients independent of PPI use. Clinicians should weigh infection risks when prescribing biologics, particularly in high-risk populations. Further studies are needed to assess risks by biologic subtype and the interplay with PPIs.

## 1. Introduction

Inflammatory bowel disease (IBD) is defined as a chronic inflammation of the gastrointestinal tract and includes two main phenotypes: Crohn’s disease (CD) and ulcerative colitis (UC). While the exact cause of this disease remains unknown, genetic susceptibility, immunological dysfunction, gut microbiota and environmental factors have been found to be implicated in its pathophysiology [1,2]. Although the disease is incurable, various available therapies aim to control disease activity and achieve biological and clinical remission. These include corticosteroids, 5-aminosalicylic acid, immunomodulators (5-a-athiopurine, methotrexate, etc.), small molecules (such as Janus kinase inhibitors and sphingosine-1-phosphate receptor inhibitors), biologics (anti-tumor necrosis factor-alpha (TNFα), anti-integrins and anti-interleukin 12/23), endoscopic and surgical interventions [3]. However, despite novel therapies, IBD continues to cause significant morbidity and complications leading to notable global, financial and psychological burden [4,5,6]. One of the main drivers of this burden is hospitalization which can occur secondary to a flare-up of the disease, with studies showing that a flare will lead to hospitalization in approximately 20% of IBD patients [6,7].

Gastrointestinal (GI) infections, caused by viruses, bacteria or parasites, are considered a common trigger for flare-ups in IBD patients [8,9]. Distinctly, IBD patients are at an increased risk of GI infections including Clostridioides difficile infections (CDI) due to immune dysregulation, gut dysbiosis and altered gut epithelial barrier [10,11]. Several studies suggest that certain IBD therapies might further increase this risk. The use of corticosteroids, immunosuppressants and biologics in IBD patients, has been linked to an increased risk of infections, including CDI [10,11,12]. However, there is a paucity of data regarding the use of biologics and the risk of non-clostridioides enteric infections.

Proton pump inhibitors (PPIs), widely used to treat gastroesophageal reflux disease and peptic ulcer disease, have been shown to increase the risk of various GI infections such as Enterovirus, Salmonella, Campylobacter and CDI [2,13,14]. This is likely due to gastric acid suppression facilitating enteric pathogen colonization and altering gut microbiota [13,15]. However, in IBD patients, the evidence is mixed. While several studies have found an increased risk of CDI in IBD patients on PPIs, others failed to reveal a statistically significant association [16,17,18,19]. Additionally, data is scarce regarding the effect of PPI use in IBD patients on the risk of non-clostridioides enteric infections with one self-controlled case series demonstrating a three-fold increased risk of enteric infections (including CDI) [20].

We conducted a retrospective study using the Northwell Health database to investigate the risk of GI infections (CDI and non-clostridioides enteric infections) in hospitalized IBD patients in relation to the use of biologics and PPIs, as well as to compare infection rates across different biologic agents. Additionally, we examined the infection type (bacterial, viral or parasitic) and the causative agents in non-clostridioides infections among the different treatment groups. Our primary objective was to estimate the odds of GI infections in hospitalized IBD patients based on treatment exposure (PPIs, biologics, both PPIs and biologics, or neither), while adjusting for demographic variables. Our secondary objectives were to: (1) Analyze the effects of the above treatment groups on the odds of CDI and non-clostridioides enteric infections, respectively, while controlling for certain demographic variables; (2) Analyze the rate of GI infections depending on the type of biologic agent used; (3) Compare infection types (bacterial, viral, parasitic) across the different treatment groups; and (4) Compare the causative agents of non-clostridioides enteric infections among the different treatment groups.

## 2. Materials and Methods

### 2.1. Data Source and Variables of Interests

We used the Northwell Inpatient Database (Northwell Health, New Hyde, NY, USA) to collect the data needed for this study, including the EPI number (a unique identifier across Northwell Health hospitals), medical record number (MRN), patient’s full name, sex, ethnicity, body mass index (BMI), IBD phenotype (UC or CD), PPI use (yes/no), use of biologic therapy (yes/no), biologic agent used (anti-TNFα: infliximab (Janssen Biotech Inc., Horsham, PA, USA)/ adalimumab (AbbVie Inc., North Chicago, IL, USA)/ golimumab (Janssen Biotech Inc., Horsham, PA, USA)/ certolizumab pegol (UCB Pharma, Brussels, Belgium), anti-integrin: vedolizumab (Takeda Pharmaceutical Company Limited, Brooklyn Park, MN, USA)/natalizumab (Biogen Idec, Cambridge, MA, USA), anti-IL-12/23: ustekinumab (Janssen Biotech Inc., Horsham, PA, USA)/risankizumab (AbbVie Inc., North Chicago, IL, USA) ), GI polymerase chain reaction (PCR) results and Clostridioides difficile (C. diff) PCR results (Fisher Scientific, Waltham, MA, USA). 

The International Classification of Disease, 10th Edition (ICD-10) codes were used to identify IBD patients (K50 for CD and K51 for UC). Patient variables of interest such as sex, ethnicity and BMI were included as covariates to control for confounders, especially that differences in age, gender and ethnicity might influence the risk of certain GI infections. 

The EPI number ensured accurate patient tracking across the Northwell Health network, preventing duplicate patients who have different MRNs due to being hospitalized in different Northwell hospitals. 

This study was approved by the Institutional Review Board (IRB: 24-0234) at Northwell Health and obtained institutional approval. 

### 2.2. Study Design

This retrospective cohort study aimed to investigate the association between GI infections and the use of PPIs and/or biologics in hospitalized IBD patients aged 18–89 years, admitted between 2013 and 2023. 

Records of patients under 18 or over 89 years of age were excluded from the study, as were duplicate records, except for patients who had a repeated GI infection when switching treatment group or biologic agent.

Our exposure of interest was the use of PPIs and/or biologics, and the study population was divided into 4 treatment groups: combined biologics and PPIs, PPIs only, biologics only and neither treatment. 

Our primary outcome was the incidence of GI infections (defined as the combination of clostridial and non-clostridial infections) among the different treatment groups. Our secondary outcomes were the evaluation of: (1) The incidence of CDI and non-clostridial infections respectively, among the different treatment groups; (2) The incidence of GI infections based on the type of biologics used; (3) The type of infection (parasitic, viral or bacterial) based on different treatment groups; and (4) The causative agent in non-clostridial infections based on different treatment groups.

C. diff PCR (and subsequent enzyme immunoassay for toxins A and B) and GI PCR were used to screen for possible CDI and non-clostridial infections respectively, with subsequent chart review to confirm an active infection and not just mere colonization. Based on the PCR results, the infection type was classified as viral, bacterial or parasitic. Charts were reviewed to confirm that IBD patients on biologics and/or PPIs were actually on the treatment prior to developing a GI infection. After appropriate data cleansing, all identifiers were removed prior to statistical analysis.

### 2.3. Statistical Analysis

Summary statistics were reported for demographics and baseline characteristics of interest. Continuous variables were presented as means with standard deviations (SD) or medians with interquartile ranges (IQR), as appropriate. Categorical variables were reported as frequencies and percentages. These statistics were computed for the entire cohort, stratified by treatment group, and by infectious colitis status (Yes vs. No). To examine differences between groups, appropriate statistical tests were used: ANOVA and two-sample *t*-tests for continuous variables, with non-parametric alternatives, Kruskal-Wallis and Wilcoxon rank sum tests, applied when assumptions of normality were violated. For categorical variables, the chi-square test or Fisher’s exact test were used.

To determine the risk of infectious colitis in IBD patients, a multivariable logistic regression model was employed, with treatment group (biologics alone, PPIs alone, both, neither) as the primary exposure variable. Additional covariates included were BMI, race/ethnicity, sex, and IBD type (CD vs UC). Model accuracy was measured using the area under the curve (AUC) c-statistic, where a statistic of 1.0 signifies near perfect accuracy, and 0.5 near perfect randomness. The following was used as a guide for AUC: 0.9–1.0 Excellent, 0.8–0.9 Very good, 0.7–0.8 Good, 0.6–0.7 Average, and 0.5–0.6 Poor. The Hosmer-Lemeshow Goodness-of-Fit test was used to test the predictive power of the model.

To assess secondary objective 1, logistic regression models were used, penalized as needed (Firth). To assess objectives 2–4, Chi-Square or Fisher’s exact tests, along with frequencies and percentages, were used.

Significance for all analyses was determined at the 0.05 level. Software utilized for data analysis was SAS Version 9.4 (SAS Institute Inc., Cary, NC, USA), R version 4.2.1 (R Foundation for Statistical Computing, Vienna, Austria), and RStudio 2024.04 (Post PBC, Boston, MA, USA).

## 3. Results

### 3.1. Demographic Distribution of IBD Patients

There were 9849 patients included in the study. The median recorded (missing: 1679) BMI in the study was 25.60 Kg/m^2^ (IQR 22.3–29.8). 45.61% (4492) of all patients were male. 67.26% (6624) of all patients were white, 10.94% (1077) were black, 8.19% (807) were Hispanic/Latino, 4.45% (438) were Asian, 6.80% (670) were multiracial or of some other race/ethnicity, and 2.37% (233) were of unknown race/ethnicity. In terms of IBD type, 42.82% (4217) of all patients had CD, and 57.18% (5632) had UC. Regarding treatment groups, 46.18% (4548) of all patients were given PPIs only, 1.17% (115) were given biologics only, 2.69% (265) were given both PPIs and biologics, and 49.96% (4921) were given neither. 1.75% (172) of all patients tested positive for GI infections, 108 by GI PCR, and 66 via the detection of Clostridioides difficile (with 2 detected by both methods) (Table 1).

There was a significant difference in BMI distributions across treatment groups (*p* < 0.0001), while significant differences in BMI distributions between infectious colitis diagnosis were not found (*p* = 0.3850). Additionally, there were found to be significant associations between race and treatment group (*p* < 0.0001), race and infectious colitis diagnosis (*p* < 0.0001), IBD type and treatment group (*p* < 0.0001), and, of interest, infectious colitis diagnosis and treatment group (*p* < 0.0001). Significant associations between sex and treatment group (*p* = 0.5945), sex and infectious colitis diagnosis (*p* = 0.8743), or IBD type and infectious colitis diagnosis (*p* = 0.4983) were not found (Table 2).

**Figure 1:**
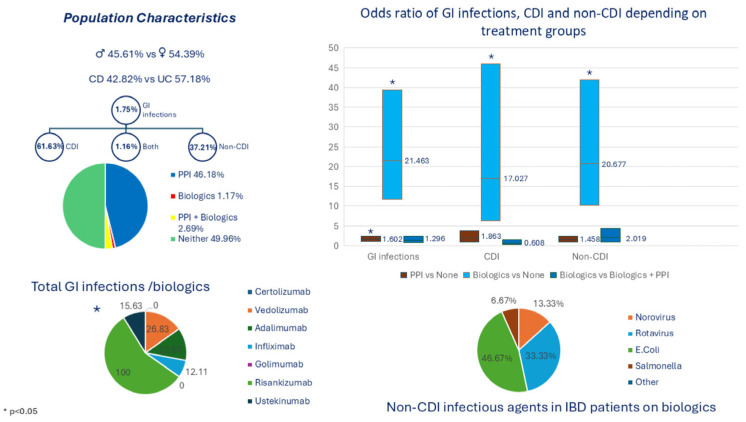
Baseline Characteristics and Key Clinical Outcomes of Hospitalized IBD Patients.

Major results were included in Figure 1.

### 3.2. Association Between Different GI Infection Categories and Treatment Groups

To assess the association of total GI infections with different treatment groups, we conducted a multivariate regression analysis, adjusting for certain demographic and clinical factors (BMI, sex, ethnicity, IBD type). Race (*p* < 0.0001) and treatment group (*p* < 0.0001) were both found to have significant effects on the prevalence of GI infection.

African American patients were 2.81 (95% confidence interval (CI) 1.87, 4.23) and 2.24 (95% CI: 1.06, 4.72) times more likely to have GI infections compared to Caucasians and Hispanics/Latinos, respectively. Additionally, multiracial patients were 2.30 (95% CI: 1.37, 3.81) times more likely to have GI infections compared to Caucasians. 

Regarding treatment categories, patients on PPI, biologics or both therapies were, respectively, 1.60 (95% CI: 1.06, 2.41), 21.46 (95% CI: 11.69, 39.40) and 16.56 (95% CI: 10.24, 26.79) times more likely to have GI infections compared to patients on no treatment. Additionally, patients on biologics alone or in combination with PPI were, respectively, 13.40 (95% CI: 7.55, 23.76) and 10.34 (95% CI: 6.68, 15.99) times more likely to have GI infections compared to patients on PPIs alone. However, no statistically significant difference was found when comparing hospitalized IBD patients on biologics only and both therapies.

The effects of BMI (*p* = 0.6939), sex (*p* = 0.7115), and IBD type (*p* = 0.5381) were all found to not have significant effects on GI infection risk (Table 3).

Regarding the association of CDI with treatment groups, a penalized Firth multivariable model showed that race (*p* < 0.0001), BMI (*p* = 0.049), and treatment group (*p* < 0.0001) were all significant predictors of CDI. Among hospitalized IBD patients, a diagnosis of CDI was more likely in African Americans (Odds ratio (OR) 6.104 with 95% CI 3.290–11.325), Asians (OR 3.198 with 95% CI 1.127–9.081) and Other/Multiracial patients (OR 4.873 with 95% CI 2.370-10.016). However, CDI was less likely in obese patients (OR 0.957, CI 0.915–1.000, *p* < 0.049, corresponding to a 0.957 reduction in the odds of CDI for every 1 Kg/m^2^ increase in BMI). Regarding treatment groups, IBD patients who were on biologics and PPI (OR 28.027, 95% CI 13.656–57.518), or biologics only (OR 17.027, 95% CI 6.299–46.022) were at a higher risk of developing CDI compared to patients on neither therapy. When comparing biologics to PPI therapy, IBD patients had a statistically significant increased risk of developing CDI if they were being treated with biologics (OR 9.138 with 95% CI 3.655–22.850). When comparing patients on both PPI and biologics to those on biologics only, or patients on PPI only to those on neither therapy, there appeared to be a trend towards an increased risk of CDI with former groups, but it was not statistically significant (Table 4).

When analyzing non-clostridial infections, a multivariable model showed that biologic treatment was associated with a higher risk of infection while neither ethnicity (*p* = 0.76) nor BMI (*p* = 0.36) were identified as risk factors in hospitalized IBD patients. IBD patients who were on biologics alone were 20.68 (95% CI: 10.20–41.90), and 14.184 (95% CI: 7.26–27.72) times more likely to have a non-clostridial infection compared to patients on no treatment and on PPI alone, respectively. IBD patients on PPIs only did not demonstrate a statistically significant risk when compared to patients not on treatment (OR 1.46, *p* = 0.13), similar to patients on biologics only compared to those on both PPIs and biologics (OR 2.02, *p* = 0.07) (Table 5).

### 3.3. GI Infections Outcomes in IBD Patients Based on Different Biologic Agents

When comparing the different biologic agents used in hospitalized IBD patients, a significant association was found between the type of biologic and GI infection status (*p* = 0.0102) (Table 6). Additionally, a similar significant association was observed between the type of biologic and non-clostridial infections (*p* = 0.001), but not CDI (*p* = 0.715).

According to this analysis (Table 6), the rates of GI, non-clostridial and CDI were 0% in patients on Certolizumab (*n* = 4) or Golimumab (*n* = 2) whereas both patients on Risankizumab had a non-clostridial infection but no CDI. Excluding these biologics, the highest rates of non-clostridial infections were seen with Vedolizumab (19.51%) followed by Ustekinumab (15.63%) and Adalimumab (10.67%) whereas the highest rates of CDI were seen with Adalimumab (12%) followed by Vedolizumab (9.76%) and Infliximab (7.62%).

### 3.4. Infection Types Categorized by Treatment Groups

There was no significant association found between treatment group and infection type (*p* = 0.8187). Only one parasitic infection was documented in our study population, occurring in IBD patients on neither therapy. The majority of GI infections were bacterial (including CDI) with rates ranging from 69.57% in the biologics-only group to 75% in patients on neither therapy, whereas viral infection ranged from 22.5% in patients on neither therapy to 30.43% in the biologics-only group. (See Table A1, Appendix A, detailing the rates of different types of GI infections in hospitalized IBD patients categorized by treatment groups).

### 3.5. Infectious Gent in Non-Clostridial Infections Categorized by Treatment Groups

When comparing hospitalized IBD patients with non-clostridial infections based on treatment groups, the majority of infections in the biologics, PPIs and neither treatment groups were bacterial while, in contrast, in the PPIs + biologics group, the majority were viral. The main bacterial agent was Escherichia Coli (33.33–46.67%) across the 4 treatment groups followed by Salmonella (6.67%) in the biologics-only group as opposed to Campylobacter (5.56–14.29%) in the remaining treatment groups. Regarding viral causative agents, the main culprit was rotavirus (33.33%) in the biologics-only group and norovirus (25.00–38.89%) in the remaining treatment groups. However, there was no statistically significant association between treatment group and infectious agent (*p* = 0.2171) (Table 7).

## 4. Discussion

Our study provides a comprehensive analysis of the associations between treatment regimens, demographic factors, and the prevalence of GI infections in IBD patients, revealing several statistically significant findings. These results align with and expand upon existing research data, offering new insights into risk stratification and management strategies for IBD patients.

### 4.1. Various Treatment Groups and Risk of GI Infections

#### 4.1.1. Biologic Therapy and Risk of GI Infections

Biologic therapies have revolutionized the treatment of IBD, but carry an inherent infection-related risk due to their immunosuppressive properties [3]. In our study, hospitalized IBD patients on biologics exhibited significantly elevated OR for GI infections compared to untreated patients: 21.46-fold higher risk of total GI infections, 20.68-fold for non-clostridial infections and 17-fold for CDI. However, the current existing literature regarding this risk remains conflicting. A systematic review and network meta-analysis by Bonovas et al. reported that biologics increased the risk of any infection (OR 1.19; 95% CI 1.10–1.29) and specifically opportunistic infections (OR, 1.90; 95% CI, 1.21–3.01) but, paradoxically, reduced the risk of serious infections [21]. Additional studies have shown that IBD patients on immunosuppressants, especially biologics, had a higher risk of common and opportunistic infections [10,12]. For example, Irving et al. observed an increased risk of both common and GI infections in IBD patients, with biologics being a significant risk factor [10].

Nevertheless, a pooled analysis by Lichtenstein et al. reported no increase in infections, serious infections, or malignancy among Infliximab-treated IBD patients compared to placebo [22]. These conflicting results underscore the complexity of assessing infection risk in IBD patients treated with biologics and highlight the importance of considering individual patient characteristics and disease severity. Notably, non-clostridial GI infection risk in biologic-treated IBD patients remains understudied. This is particularly of interest because enteral infections have been linked to IBD flares, significant morbidity and mortality [8,23,24].

Similarly, several studies showed worse outcomes in IBD patients with CDI, including increased infection severity, prolonged hospitalization, and higher mortality [11,17,25]. However, the relationship between the use of biologics in these patients and CDI risk remains controversial. A systematic review and meta-analysis of 22 studies by Balram et al. reported that biologics use was associated with an increased risk of CDI in IBD patients (OR: 1.65, 95% CI: 1.18, 2.30), similarly to our study findings [18]. On the other hand, the RECIDIVISM Study showed that only infliximab was associated with an increased risk of recurrent CDI while another study reported a higher risk of CDI solely with the sequential use of 3 or more biologic agents [17,26]. Additionally, other studies did not find any association between biologic treatment and CDI in IBD patients [16,19,26,27]. These discrepancies likely stem from differences in study design, baseline disease severity, or unmeasured confounders. As an example of confounders, the concomitant use of corticosteroids, other immunosuppressive agents (such as thiopurines and methotrexate), and antibiotics is particularly important. These medications are frequently used in IBD management and can independently increase the risk of gastrointestinal infections through immunosuppression or disruption of the gut microbiota, potentially confounding the observed associations between biologic therapy and infection risk. Belram et al. reported a significant association between CDI and antibiotic use within 30 days (OR 1.85, 95% CI 1.36–2.52), but found no association with corticosteroid or immunosuppressant use [18]. In contrast, another study demonstrated a two-fold increased risk of CDI with immunomodulators (such as 6-mercaptopurine, azathioprine, and methotrexate) and a three-fold increased risk within 90 days of corticosteroid initiation [28]. Similarly, Irving et al. identified a link between CDI and both immunotherapy and corticosteroid use [10]. In our study, we did not control for concomitant use of these medications, which may have influenced the associations observed.

One important point worth highlighting is that increased disease activity has been associated with a higher risk of infections [29,30]. A nationwide Swedish study found that IBD patients with histologic inflammation had a significantly greater risk of serious infections, including GI infections, compared to those in remission [31]. Similarly, Vitikainen et al. identified active IBD as a major risk factor for CDI [32]. In our analysis, we were unable to account for baseline disease activity prior to hospitalization, which may have influenced the associations observed. This limitation is particularly relevant, as patients receiving biologic therapy often have more severe disease at baseline. However, it is also important to note that biologic therapy is associated with higher rates of clinical, endoscopic, and histologic remission, including mucosal healing [33,34].

Moreover, obese IBD patients had a lower risk of CDI with a 0.957 reduction in the odds of infection for every 1 Kg/m^2^ increase in BMI. It is unclear how BMI can affect infection risk in IBD patients but one explanation could be a potential association between disease severity and BMI. Some studies suggested that obese patients with CD had a reduced risk of penetrating disease, hospitalizations, CD-related surgery and the need for anti-TNF therapy while others, such as Greuter et al., found obesity to be associated with an increased risk of complications and decreased remission [35,36]. In ulcerative colitis, the evidence is similarly mixed, with some studies reporting worse disease outcomes among obese patients, while others do not demonstrate a clear correlation [35]. This variability could be attributed to the use of BMI to define obesity. Emerging data indicate that BMI alone may not be accurate in assessing obesity as it does not differentiate between visceral and subcutaneous adiposity. In particular, visceral adipose tissue has been more closely associated with disease activity, systemic inflammation, and complications in IBD [35]. In our analysis, while BMI was included as a potential confounder, we acknowledge the limitation of not having data on body fat distribution or objective disease activity measures, which could have further informed our interpretation of the observed associations.

#### 4.1.2. PPI Therapy and Risk of GI Infections

In our cohort, PPI use alone was associated with a 1.6-fold increased risk of total GI infections compared to no treatment (Table 3). While trends toward higher CDI and non-clostridial infection risks were observed, these did not reach statistical significance (Table 4 and Table 5), potentially due to small subgroup sample sizes or unadjusted confounders.

These findings align with the available literature linking the use of PPI to an increased risk of enteric infections, though the magnitude of the risk and pathogen type vary across studies. A systematic review and meta-analysis by Bavishi and Dupont established that PPIs increased the risk of various enteric infections, such as CDI, Salmonella, and Campylobacter, in the general population [14]. The mechanism behind this association is thought to involve PPI-induced reduction in gastric acid production, gut microbiota disruption and enhanced pathogen colonization [14,37]. Similarly, other studies showed increased acute gastroenteritis risks, particularly during high viral circulation periods, in addition to higher hospitalization rates for infectious gastroenteritis among PPI users [13,38]. However, Brophy et al. argued that patient demographics-not PPIs-primarily drive the infection risk [39]. In IBD populations, evidence remains limited, though a recent self-controlled case series found a three-fold increased risk of enteric infections during PPI exposure with C. diff and Escherichia coli (*E. coli*) as predominant pathogens [20]. For CDI specifically, several studies including meta-analyses showed an increased risk with PPI use in the general population, while this has been less consistently demonstrated in IBD patients [40,41,42,43]. Stoica et al. and Li et al. found significant CDI risks with PPI use (OR = 1.57 and 2.39, respectively), whereas Balram et al. found no association [16,18,44].

#### 4.1.3. Combination of Biologics and PPI Therapy and Risk of GI Infections

While existing literature explores, to a certain extent, the individual risks of biologics and PPIs on GI infections, no prior studies have examined their combined effect in IBD patients, a gap our study addresses by revealing nuanced associations between these therapies and various enteric infections. 

Specifically, PPI/biologic co-therapy was associated with significantly higher risks of overall GI infections, CDI, and non-clostridial infections compared to patients receiving neither treatment. When directly comparing co-therapy to PPIs alone, risks for all infection types were statistically significantly increased with cotherapy, whereas no significant risk differences emerged between co-therapy and biologics alone. In fact, biologics-only patients showed non-significant trends toward higher GI (OR 1.3) and non-clostridial infection risks (OR 2.02) but lower CDI risk (OR 0.61) compared to co-therapy patients. Additionally, biologics alone posed higher GI, CDI and non-clostridial infection risks than PPIs alone. These findings seem to suggest that biologic therapy is likely the primary drivers of enteric infection risk while the concomitant usage of PPIs didn’t significantly affect that risk. 

One potential explanation for this observation is that biologics, through their immunosuppressive mechanisms, severely impair the host’s ability to control enteric pathogens [45]. Although PPIs can disrupt the gut microbiome and reduce gastric acidity, potentially facilitating bacterial colonization and CDI, our results suggest this effect may be attenuated in patients already on biologics [46]. In other words, immune suppression from biologics creates a permissive environment for infections, which, when combined with the baseline infection risk inherent to IBD, may render PPI-related effects non-significant. However, it’s essential to acknowledge that these findings are based on a retrospective study, necessitating validation through larger, prospective studies which could clarify whether PPIs paradoxically mitigate GI and non-clostridial infection risks when combined with biologics. 

Regardless, PPI use in combination with biologics should be carefully considered as one meta-analysis linked PPIs to reduced remission rates with infliximab, while a Hungarian nationwide cohort study reported similar findings with vedolizumab [47,48]. Moreover, several studies reported worse IBD related outcomes such as hospital admission, surgery and initiation of new biologic therapy in IBD patients on PPIs [46,47,49]. Thus, unless a clear indication exists, PPIs should be avoided in biologic-treated IBD patient to minimize complications and treatment failure.

### 4.2. GI Infection Risk with Various Biologic Agents

Our study revealed significant variations in GI infection risks among IBD patients receiving different biologic agents, with vedolizumab associated with highest overall GI infection risk, followed by adalimumab, and ustekinumab. While the risk of CDI was numerically highest with adalimumab, this difference did not reach statistical significance, reflecting heterogeneity in existing literature. For example, Dalal et al. reported no significant CDI risk difference between vedolizumab and anti-TNFα in UC patients (hazard ratio (HR) 0.33, 95% CI 0.05–2.03), but identified lower severe CDI risk with vedolizumab (HR 0.10, 95% CI 0.01–0.76) [50]. Similarly, a meta-analysis of 30 studies by Chen et al. showed a higher CDI risk in UC patients receiving vedolizumab compared to those with CD, which implicates that Vedolizumab could potentially be offering an advantage over anti-TNF agents for UC regarding CDI risk, but not for CD [51]. Our findings extend the understanding of infection risks across a broader spectrum of biologics, including ustekinumab and risankizumab. The higher overall GI infection risk with vedolizumab, particularly for non-clostridial infections, may be attributed to its gut-selective mechanism of action, which could potentially alter local mucosal immunity [52]. The trend towards higher CDI risk with adalimumab, although not statistically significant, warrants further investigation, as it contrasts with some existing literature on anti-TNFα agents. Furthermore, ustekinumab’s lower infection risk profile in our cohort is an intriguing finding that requires validation in larger studies while the observed 100% risk of GI and non-clostridial infections with risankizumab is likely due to the very small sample size (*n* = 2).

These results, though limited by sample cohorts particularly for newer agents like ustekinumab and risankizumab, underscore the complex interplay between biologic mechanisms and infection susceptibility in IBD patients. It is important to interpret these findings with caution. First, our study reports the cumulative incidence of infections over a 10-year period, rather than the annualized infection rates commonly presented in clinical trials and large cohort studies. For instance, long-term safety analysis of adalimumab in CD and UC reported serious infection rates of approximately 3.5–6.9% per year [53,54]. Similarly, a Swedish cohort study of IBD patients on Vedolizumab reported GI infection incidence rates of 1.97% per year (95% CI 1.22–3.02) in UC and 1.41% per year (95% CI 0.84–2.23) in CD, comparable to placebo [55]. Over a 10-year span, the cumulative risk for patients continuously exposed to biologics could approach 10–40%, assuming a constant annual risk and no competing events. As such, the higher infection percentages observed in our study reflect cumulative incidence over multiple years and should not be directly compared to the annualized rates reported in clinical trials. Second, our study was not designed to determine the absolute risk of infection attributable to each specific biologic agent. Rather, our primary objective was to compare infection rates across treatment groups in a hospitalized IBD population. The relatively small sample sizes for individual biologics, the retrospective study design, and the lack of precise exposure duration for each biologic agent limit our ability to draw firm conclusions about the infection risk associated with any one therapy.

### 4.3. Infectious Type and Agents Across Treatment Groups

Our study examined the association between treatment groups and infection types in hospitalized IBD patients, finding no statistically significant link (*p* = 0.8187) but providing valuable insights into the patterns of infections across different therapies. Bacterial infections, including CDI, were predominant across all treatment groups, which is consistent with prior studies showing higher rates of bacterial pathogens in IBD-associated GI infections. For example, Axelrad et al. identified bacterial pathogens in 66.4% of CD and 82.1% of UC patients with GI infections, while another study reported CDI in 63.5% and *E. coli* in 27% of IBD patients during flares [23,56]. In our study, non-clostridial infections were primarily caused by *E. coli* followed by Salmonella or Campylobacter, depending on the treatment group. This is consistent with the few studies present in the literature and is intriguing as certain bacterial pathogens like Campylobacter, have been linked to worse IBD flare outcomes, including higher colectomy and mortality rates [23,57]. Viral infections, though less common, were mainly attributable to norovirus and rotavirus, mirroring patterns observed in both IBD populations and the general public [23,58].

While our results showed no significant associations between treatment and infection types or causative agents, these observations provide valuable insights into the complex interplay between IBD therapies and infection risk. Additionally, the lack of statistical significance may be due to the relatively small sample size, particularly in the biologics-only group, and highlights the need for larger, prospective studies to further elucidate these relationships.

Our study has several limitations. Its retrospective nature limits the deductions that can be made from the associations. The use of ICD-10 codes to define our study population is dependent on accurate documentation and diagnosis and could be prone to errors. We did not include certain factors such as age, socioeconomic background or rural/urban distribution in our analysis which could act as confounders. Additionally, the exclusion of disease activity measures, endoscopic findings, and concomitant use of steroids or immunosuppressants further limits the ability to fully assess the impact of treatment groups on GI infections. Another limitation is that the exact timing of biologic initiation relative to GI infection onset could not be determined, as all patients had started biologic therapy prior to hospitalization, limiting our ability to assess time-dependent infection risk. Furthermore, our study population consisted exclusively of hospitalized IBD patients, which might not be truly representative of the entire IBD population, notably because hospitalized patients are likely to have more severe disease or be experiencing a flare-up. Additionally, the proportion of IBD patients on biologic therapy in our cohort was lower than typically reported in large US epidemiological studies [59]. This likely reflects the fact that our hospital is not a specialized IBD center and does not have dedicated IBD specialists, resulting in a patient population with milder disease who are less likely to require biologic therapy. Patients with more severe or refractory disease are often referred to tertiary centers where biologic use is more prevalent. Furthermore, real-world treatment patterns often involve a step-up approach, and some patients in our cohort may not have yet escalated to biologic therapy at the time of the study. Insurance and access barriers, as well as patient and provider preferences, may also contribute to delayed or reduced biologic use in non-specialist settings. Therefore, our findings regarding biologic use should not be interpreted as representative of the broader US IBD population. Finally, we included duplicate records of IBD patients who had recurrent GI infections if they changed treatment groups or biologic agents which might be a confounding factor.

## 5. Conclusions

In conclusion, biologic therapy was significantly associated with elevated risks of overall GI, CDI and non-clostridial infections in IBD patients, while PPI use correlated with increased overall GI infection risk. These findings, when considered alongside existing literature, highlight the need for thoughtful prescribing practices in IBD management, particularly given the association between GI infections and worse outcomes in IBD patients including higher hospitalization rates, elevated disease severity scores and reduced quality of life [25,60].

While our study identifies key associations warranting further investigation, larger prospective studies are crucial to confirm these findings and clarify underlying mechanisms. This research underscores the importance of individualized treatment strategies in IBD, carefully weighing the potential infectious risks and other adverse outcomes associated with biologics and PPIs over the therapeutic benefits.

## Figures and Tables

**Table 1:** Baseline characteristics in the study population.

Characteristic		
Study Population (n)		9849
Sex (%)		
	Male	45.61%
	Female	54.39%
Race/Ethnicity (%)		
	White	67.26%
	African American	10.94%
	Hispanic/Latino	8.19%
	Asian	4.45%
	Multiracial or other	6.80%
	Unknown	2.37%
Body Mass Index (Kg/m^2^)		
	Median (IQR)	25.60 (22.3–29.8)
Type of IBD (%)		
	Crohn’s Disease	42.82%
	Ulcerative Colitis	57.18%
Treatment Groups (%/n)		
	PPIs Only	46.18% (4548)
	Biologics Only	1.17% (115)
	Both PPIs and Biologics	2.69% (265)
	Neither PPIs nor Biologics	49.96% (4921)
GI Infection (%/n)		
	No	98.25% (9677)
	Yes	1.75% (172)
	a—Detected by GI PCR Only	61.63% (106)
	b—Detected C. diff PCR Only	37.21% (64)
	c—Detected by both	1.16% (2)

C. diff: Clostridioides difficile, GI: gastrointestinal, IQR: interquartile range, PCR: polymerase chain reaction, PPI: proton pump inhibitors.

**Table 2:** Demographic and clinical characteristics in IBD patients among different treatment groups.

Characteristic		Biologics	Biologics +PPIs	None	PPIs	*p*-Value
Sex (%)						*p* = 0.5945
	Male	1.19%	2.69%	50.72%	45.40%	
	Female	1.14%	2.69%	49.07%	47.11%	
Race/Ethnicity (%)						*p* < 0.0001 *
	African American	1.49%	3.99%	45.68%	48.84%	
	Asian	2.51%	2.05%	46.35%	49.09%	
	Caucasian	1.06%	2.45%	50.53%	45.97%	
	Hispanic/Latino	0.25%	0.74%	48.70%	50.31%	
	Other/Multiracial	2.24%	6.12%	54.18%	37.46%	
Body Mass Index (Kg/m^2^)						*p* < 0.0001 *
	Median (IQR)	23.35 (20.30–26.70)	26.10 (21.40–29.30)	25.40 (22.10–29.60)	25.80 (22.50–30.00)	
Type of IBD (%)						*p* < 0.0001 *
	Crohn’s Disease	1.38%	3.53%	49.28%	45.81%	
	Ulcerative Colitis	1.01%	2.06%	50.48%	46.45%	
GI infection (%/n)						*p* < 0.0001 *
	No	0.95%	2.33%	50.44%	46.29%	
	Yes	13.37%	23.26%	23.26%	40.12%	

* *p* < 0.05, considered statistically significant. (GI: gastrointestinal, IQR: interquartile range, PPI: proton pump inhibitors).

**Table 3:** Association between GI infections and treatment groups in hospitalized IBD patients.

Odds Ratio Estimates				
Effect	Point Estimate	95% Wald Confidence Limits		*p*-Value
BMI	0.995	0.970	1.021	0.6939
Female vs. Male	1.064	0.766	1.479	0.7115
African American vs. Caucasian	2.814	1.872	4.231	<0.0001 *
Asian vs. Caucasian	1.679	0.815	3.457	0.1599
Hispanic/Latino vs. Caucasian	1.259	0.621	2.550	0.5231
Other/Multiracial vs. Caucasian	2.304	1.396	3.805	**0.0011** *
CD vs. UC	0.901	0.646	1.256	0.5381
Biologics vs. Biologics + PPIs	1.296	0.699	2.401	0.4114
Biologics vs. None	21.463	11.693	39.396	<0.0001 *
Biologics vs. PPIs	13.396	7.551	23.764	<0.0001 *
Biologics + PPIs vs. None	16.561	10.239	26.786	<0.0001 *
Biologics + PPIs vs. PPIs	10.336	6.682	15.989	<0.0001 *
PPIs vs. None	1.602	1.064	2.413	0.0241 *
Hosmer-Lemeshow Test	*p* = 0.8395			
*c*-statistic	0.740			

* *p* < 0.05, considered statistically significant. (BMI: body mass index, CD: Crohn’s disease, GI: gastrointestinal, IBD: inflammatory bowel disease, PPI: proton pump inhibitors, UC: ulcerative colitis).

**Table 4:** Association between CDI and treatment groups in hospitalized IBD patients.

Odds Ratio Estimates and Wald Confidence Intervals				
Effect	Point Estimate	95% Confidence Limits		*p*-Value
African American vs. Caucasian	6.104	3.290	11.325	<0.0001 *
Asian vs. Caucasian	3.198	1.127	9.081	0.029 *
Hispanic/Latino vs. Caucasian	2.684	0.953	7.558	0.062
Other/Multiracial vs. Caucasian	4.873	2.370	10.016	<0.0001 *
BMI	0.957	0.915	1.000	0.049 *
Biologics vs. Biologics + PPIs	0.608	0.241	1.533	0.2924
Biologics vs. None	17.027	6.299	46.022	<0.0001 *
Biologics vs. PPIs	9.138	3.655	22.850	<0.0001 *
Biologics + PPIs vs. None	28.027	13.656	57.518	<0.0001 *
Biologics + PPIs vs. PPIs	15.042	8.200	27.593	<0.0001 *
PPIs vs. None	1.863	0.929	3.737	0.080
Hosmer-Lemeshow Test	*p* = 0.4546			
*c*-statistic	0.821			

* *p* < 0.05, considered statistically significant. (BMI: body mass index, CDI: clostridioides difficile infection, IBD: inflammatory bowel disease, PPI: proton pump inhibitors).

**Table 5:** Risk of non-clostridial infections in hospitalized IBD patients according to treatment groups.

Odds Ratio Estimates				
Effect	Point Estimate	95% Wald Confidence Limits		*p* Value
African American vs. Caucasian	1.513	0.870	2.632	0.143
Asian vs. Caucasian	1.211	0.474	3.093	0.689
Hispanic/Latino vs. Caucasian	0.865	0.343	2.177	0.757
Other/Multiracial vs. Caucasian	1.255	0.624	2.522	0.524
BMI	1.014	0.984	1.045	0.357
Biologics vs. Biologics + PPIs	2.019	0.944	4.315	0.071
Biologics vs. None	20.677	10.204	41.900	<0.0001 *
Biologics vs. PPIs	14.184	7.259	27.718	<0.0001 *
Biologics + PPIs vs. None	10.243	5.503	19.063	<0.0001 *
Biologics + PPIs vs. PPIs	7.026	3.941	12.527	<0.0001 *
PPIs vs. None	1.458	0.889	2.389	0.135
Hosmer-Lemeshow Test	*p* = 0.4131			
*c*-statistic	0.689			

* *p* < 0.05, considered statistically significant. (BMI: body mass index, CD: Crohn’s disease, GI: gastrointestinal, IBD: inflammatory bowel disease, PPI: proton pump inhibitors, UC: ulcerative colitis).

**Table 6:** Rates of total GI, non-clostridial and clostridioides difficile infections in hospitalized IBD patients depending on different biologic agents and subsequent association.

Biologic Therapy	Number of IBD Patients on Treatment	Any GI Infection (%)	Non-Clostridial Infection (%)	CDI (%)
Certolizumab	4	0.00	0.00	0.00
Vedolizumab	41	26.83	19.51	9.76
Adalimumab	75	22.67	10.67	12.00
Infliximab	223	12.11	4.93	7.62
Golimumab	2	0.00	0.00	0.00
Risankizumab	2	100.00	100.00	0.00
Ustekinumab	32	15.63	12.50	3.13
*p* value		0.0102 *	0.001 *	0.715

* *p* < 0.05, considered statistically significant. (CDI: clostridioides difficile infection, GI: gastrointestinal, IBD: inflammatory bowel disease).

**Table 7:** Infectious agent rates in IBD patients with non-clostridial infections based on treatment groups (*p* = 0.2171).

Treatment	Biologics (n) Perc. (%)	PPIs (n) Perc. (%)	Biologics+PPIs (n) Perc. (%)	None (n) Perc. (%)
Adenovirus	0	0	0	1
	0.00%	0.00%	0.00%	3.57%
Astrovirus	0	1	1	1
	0.00%	2.13%	5.56%	3.57%
Norovirus	2	15	7	7
	13.33%	31.91%	38.89%	25.00%
Rotavirus	5	4	2	0
	33.33%	8.51%	11.11%	0.00%
Sapovirus	0	0	1	0
	0.00%	0.00%	5.56%	0.00%
*E. coli*	7	16	6	12
	46.67%	34.04%	33.33%	42.86%
Campylobacter	0	6	1	4
	0.00%	12.77%	5.56%	14.29%
Salmonella	1	4	0	1
	6.67%	8.51%	0.00%	3.57%
Yersinia	0	1	0	1
	0.00%	2.13%	0.00%	3.57%
Giardia	0	0	0	1
	0.00%	0.00%	0.00%	3.57%
Total	15	47	18	28

*E. coli*: Escherichia coli, IBD: inflammatory bowel disease, Perc.: percentage, PPIs: proton pump inhibitors.

## Data Availability

The datasets presented in this article are not readily available due to concern for privacy/ethics. Requests to access the datasets should be directed to Northwell’s IRB.

## Abbreviations

The following abbreviations are used in this manuscript:

AUC	Area under the curve
BMI	Body mass index
CD	Crohn’s disease
CDI	Clostridioides difficile infections
C.diff	Clostridioides difficile
CI	Confidence interval
GI	Gastrointestinal
IBD	Inflammatory bowel disease
ICD-10	International Classification of Disease, 10th Edition
IQR	Interquartile ranges
IRB	Institutional Review Board
MRN	medical record number
OR	Odds ratio
PCR	Polymerase chain reaction
PPI	Proton pump inhibitor
TNFα	Tumor necrosis factor-alpha
UC	Ulcerative colitis

## Appendix A

**Table A1:** Rates of different types of GI infections in hospitalized IBD patients categorized by treatment groups. (*p* = 0.8187). (GI: gastrointestinal, IBD: inflammatory bowel disease, PPI: proton pump inhibitors).

	Infection Type			
Treatment	Bacterial	Parasite	Viral	Total
Biologics	16 (69.57%)	0 (0.00%)	7 (30.43%)	23
PPIs	49 (71.01%)	0 (0.00%)	20 (28.99%)	69
Biologics + PPIs	29 (72.50%)	0 (0.00%)	11 (27.50%)	40
None	30 (75.00%)	1 (2.50%)	9 (22.50%)	40
Total	124	1	47	172
